# Affected Sib-Pair Analyses Identify Signaling Networks Associated With Social Behavioral Deficits in Autism

**DOI:** 10.3389/fgene.2019.01186

**Published:** 2019-11-27

**Authors:** Mehdi Pirooznia, Tejasvi Niranjan, Yun-Ching Chen, Ilker Tunc, Fernando S. Goes, Dimitrios Avramopoulos, James B. Potash, Richard L. Huganir, Peter P. Zandi, Tao Wang

**Affiliations:** ^1^Bioinformatics and Computational Biology Core Facility, National Heart Lung and Blood Institute, NIH, Bethesda, MD, United States; ^2^Department of Psychiatry and Behavioral Sciences, Johns Hopkins University School of Medicine, Baltimore, MD, United States; ^3^McKusick-Nathans Department of Genetic Medicine, Johns Hopkins University School of Medicine, Baltimore, MD, United States; ^4^Department of Neuroscience, Johns Hopkins University School of Medicine, Baltimore, MD, United States; ^5^Department of Mental Health and Epidemiology, Johns Hopkins University School of Public Health, Baltimore, MD, United States

**Keywords:** affected sibs, autism social behavior, network analysis, InWeb_IM, GeNetMeta, synaptome, PI3K-AKT-mTOR, NGF signaling

## Abstract

Autism spectrum disorders (ASDs) are characterized by deficits in three core behavioral domains: reciprocal social interactions, communication, and restricted interests and/or repetitive behaviors. Several hundreds of risk genes for autism have been identified, however, it remains a challenge to associate these genes with specific core behavioral deficits. In multiplex autism families, affected sibs often show significant differences in severity of individual core phenotypes. We hypothesize that a higher mutation burden contributes to a larger difference in the severity of specific core phenotypes between affected sibs. We tested this hypothesis on social behavioral deficits in autism. We sequenced synaptome genes (n = 1,886) in affected male sib-pairs (n = 274) in families from the Autism Genetics Research Exchange (AGRE) and identified rare (MAF ≤ 1%) and predicted functional variants. We selected affected sib-pairs with a large (≥10; n = 92 pairs) or a small (≤4; n = 108 pairs) difference in total cumulative Autism Diagnostic Interview-Revised (ADI-R) social scores (SOCT_CS). We compared burdens of unshared variants present only in sibs with severe social deficits and found a higher burden in SOCT_CS≥10 compared to SOCT_CS ≤ 4 (SOCT_CS≥10: 705.1 ± 16.2; SOCT_CS ≤ 4, 668.3 ± 9.0; *p* = 0.025). Unshared SOCT_CS≥10 genes only in sibs with severe social deficits are significantly enriched in the SFARI gene set. Network analyses of these genes using InWeb_IM, molecular signatures database (MSigDB), and GeNetMeta identified enrichment for phosphoinositide 3-kinase (PI3K)-AKT-mammalian target of rapamycin (mTOR) (Enrichment Score [eScore] *p* value = 3.36E−07; n = 8 genes) and Nerve growth factor (NGF) (eScore *p* value = 8.94E−07; n = 9 genes) networks. These studies support a key role for these signaling networks in social behavioral deficits and present a novel approach to associate risk genes and signaling networks with core behavioral domains in autism.

## Introduction

Autism spectrum disorders (ASDs) are a group of heterogeneous neurodevelopmental disorders characterized by deficits in reciprocal social interactions, communication, and restricted interests and/or repetitive behaviors. ASDs are caused by a combination of environmental risk factors and genetic mutations (Devlin and Scherer, 2012; Huguet et al., 2013). Twin-, family-, and population-based studies indicate that genetic factors contribute to more than half of the risk of developing ASDs (Klei et al., 2012; Gaugler et al., 2014; Sandin et al., 2014). A recent meta-analysis on 6,413 twins including affected twins showed that heritability in families with an autistic patient is 64–91% (Tick et al., 2016). Common variants of small effect and rare variants of large effect could have a substantial impact on risk of developing autism and/or on severity of specific behavioral domains in autism (Huguet et al., 2013).

Genome-wide sequencing of large cohorts of autism patients and their families has generated considerable numbers of sequence variants in recent years (Michaelson et al., 2012; Lim et al., 2013; Yu et al., 2013). Case-control and family-based studies implicate risk alleles by identifying an association of common variants of small effect, a higher mutation load of rare variants of large effect, and/or presence of *de novo* variants in affected probands (De Rubeis and Buxbaum, 2015). Several hundreds of autism risk genes have been implicated using these approaches (Huguet et al., 2013). However, the majority of these studies treat autism as a “single disease” in a “case-control” study design rather than a spectrum of disorders with deficits in core behavioral domains. Diagnosis of ASDs are based on standard, semi-quantitative behavioral tests including Autism Diagnostic Interview-Revised (ADI-R) (Lord et al., 1994) to assess deficits in three core behavioral domains: reciprocal social interaction, communication, and restricted interests and/or repetitive behaviors. Although defects in all three domains are required to make a diagnosis, patients with ASDs often present with significant differences in the severity of specific behavioral domains. Furthermore, deficits in these behavioral domains are shared by other neuropsychiatric disorders. For example, abnormal social behaviors are a key feature of schizophrenia; increased repetitive behaviors or movements are a core phenotype in obsessive-compulsive disorder, and defects in speech and communication are seen in neurodevelopmental syndromes with different underlying genetic causes. Understanding shared mechanisms responsible for specific domains of psychiatric phenotypes in these disorders is crucial to development of medications and interventions for individualized care for patients with these disorders and identification of family members at increased risk for genetically influenced behavioral or psychiatric phenotypes. Due to extensive genetic heterogeneity and phenotypic variability (Jeste and Geschwind, 2014; De Rubeis and Buxbaum, 2015), it remains a major challenge to associate genetic risk genes and networks with specific behavioral domains in autism.

It has long been noted that in multiplex families, affected sibs with autism often show a significant difference in the severity of one or more behavioral domains (Spiker et al., 2002). We hypothesize that genetic burdens of cumulative risk genes and signaling networks contribute to the differences in severity of specific domains in the affected sibs. A recent WGS study of a cohort of quartet autism families showed that in a large fraction (50–69%) of multiplex families, two affected sibs do not share the same rare penetrant risk alleles (Yuen et al., 2015). These affected sibs with discordant mutations tended to demonstrate more phenotypic variability as compared to those who shared the same risk variants. These results support a strong genetic determinant responsible for differences in severity of specific autism phenotypes between affected sibs.

Genetic studies of affected sib-pairs have been used previously to map genes for rare Mendelian disorders based on the principle of identical-by-descent (IBD) (Zhang and Risch, 1996; Liang et al., 2001; Perdry et al., 2012). It has been shown that an affected sib-pair design based on sharing pathogenic variants allows a tremendous gain of analysis power over a traditional case-control study design to implicate pathogenesis of rare variants (Xing et al., 2006; Sul et al., 2017). Discordant sib-pair designs have also been used in genetic linkage and association studies to increase power of analysis (Boehnke and Langefeld, 1998; Poznik et al., 2006). We explore a novel family-based strategy utilizing affected, phenotypic discordant sib-pairs to identify rare genetic variants of large effect contributing to specific domains in autism (Hu and Steinberg, 2009; Hu et al., 2011; Sacco et al., 2012; Veatch et al., 2014). We hypothesize that (Huguet et al., 2013) affected sibs with severe deficits in specific domains carry a larger burden of risk variants as compared to sibs with mild deficits in the same domain, (Devlin and Scherer, 2012) differences in severity of deficits in specific domains by ADI-R scores between affected sibs correlate with burden of cumulative risk variants, and (Gaugler et al., 2014) sets of autism risk genes associated with specific autism domain(s) in one family may be shared by a fraction of affected families in a study cohort.

In the current study, we sequenced the exons of synaptome genes (n = 1,886) in 274 pairs of affected male siblings from Autism Genetics Research Exchange (AGRE). We identified an excess burden of rare deleterious variants in cohorts of sibs showing a large versus small differences in severity of social deficits defined by total cumulative ADI-R social interaction score (SOCT_CS). We performed network-based analyses on these gene sets carrying the excess mutations and identified several neural signaling networks associated with social behavioral deficits in autism.

## Methods

### Patients

We surveyed autism pedigrees in the AGRE repository and identified 274 pairs of male affected sibs in multiplex families (www.autismspeaks.org/agre). We selected male affected sibs who have full behavioral evaluations including ADI-Rs and show significant differences in the severity of autism phenotypes, i.e., one sib presents as severe while the other sib presents mild phenotype as defined by the cumulative ADI-R scores. Three-generation pedigrees, DNA samples, developmental histories, and behavioral test scores including ADI-R and ADOS are obtained for all enrolled patients and most relatives in these families. An institutional review board at the Johns Hopkins University has approved this study.

### Synaptome Analysis

#### Sequencing

We surveyed all published proteomics studies and publically available databases that focus on the synapse and identified genes that encode 1,886 synaptic proteins consisting of proteins found in the vesicles (N = 107), in the presynaptic membrane (N = 336), in the presynaptic active zone (N = 209) and in the post-synaptic density, as established in SynaptomeDB (http://metamoodics.org/SynaptomeDB/) (Pirooznia et al., 2012). We utilized an Agilent Sure-Select target enrichment kit to capture 6.7 Mb of targeted genomic sequence for human Synaptome and completed next-generation sequencing for 274 affected sib-pairs and 336 matched normal controls using HiSeq2000 at the high-throughput sequencing core at the Johns Hopkins University (Pirooznia et al., 2016).

#### Data Processing

Sequence reads were aligned to the human reference genome (UCSC hg19) using BWA aligner (Li and Durbin, 2009) allowing for two mismatches in the 30-base seed. Picard (http://picard.sourceforge.net/) was used to fix any mate pair mismatch and remove reads with identical outer mapping coordinates, which represent likely PCR artifacts. Target coverage for the Agilent Sure Select capture was assessed using Picard’s HSmetrics utility. The Genome Analysis Toolkit (Mckenna et al., 2010) was used to generate SNV and small indel calls within the targeted regions. We performed variant calling using GATK’s HaplotypeCaller followed by a Variant Recalibration step. SNV clusters, defined as greater than three SNVs per ten bases, and SNVs falling within a called indel region, were masked. Variant Call Format (VCF) files were converted to PLINK file format using VCF tools and custom scripts. PLINK was subsequently used to remove variants with >10% missing calls and variants in Hardy-Weinberg Disequilibrium (*p* < 1 × 10^−6^). Principal component analysis (PCA) of the case-control sample was performed using Eigenstrat to assess for potential population stratification and batch effects across the sequencing platforms using common sequenced variants (MAF > 0.05) pruned to be in approximate linkage equilibrium. We inspected the top axes of variation in each PCA component and removed three outlier individuals, with the remaining samples showing appropriate clustering consistent with a European-American sample.

#### Annotation

Identified variants, including single nucleotide variants (SNVs) and indels, were annotated with ANNOVAR (Wang et al., 2010) using Ensembl release version 63 as the reference assembly. ANNOVAR provides information on gene annotation, amino acid change annotation, dbSNP ids, 1000 Genomes Project allele frequencies, and NHLBI-Exome Sequencing Project (ESP) allele frequencies. For annotation of missense variants, we used SIFT and Polyphen-2 to identify variants of potentially damaging effect. We used default thresholds of SIFT (>0.95) and PolyPhen (>0.85) to classify a SNV as damaging. For indels, we included stopgain, stoploss, frameshift, and splicing insertions as damaging variants. Rare variants, defined as having a population frequency ≤1% were selected for gene burden analyses by using the European-American and ALL frequency estimates of the NHLBI-ESP, and both the European-American and ALL estimates from the 1000 Genomes April 2012 release to exclude variants with allele frequency >1% in any of these external datasets.

### Pathway Analysis

We employed GeNets platform (Li et al., 2018) for network and pathway analyses to evaluate the connectivity of genes in our gene-set, based on a network of known susceptibility genes that are interconnected by protein–protein interaction (PPI) using InWeb. It builds “neighborhoods” of genes in a gene list that are more interconnected within the reference network by creating a general classifier to predict membership from networks in the InWeb PPI network. For any candidate gene, the classifier can assign a probability that it belongs to a pathway as defined by the candidate’s architectural properties in the overall network. GeNets creates networks of PPIs using evidence of physical interaction from the InWeb database, which contains more than 420,000 high-confidence pair-wise interactions involving 12,793 proteins (Lage et al., 2007; Wagner et al., 2012; Li et al., 2017; Raj et al., 2018). It displays these interaction networks as community structures (also called modular sub-network structure). A module is a set of genes (called nodes) that are more connected to one another than they are to other groups of genes based on a probability score that is calculated based on network metrics using a machine learning algorithm (quack) trained on 853 curated molecular signatures database (MSigDB) pathways with the reference network; and using that same algorithm to “predict” other genes in the network that are not on our gene list, but may belong to the same pathway that is captured by our gene list. The 853 MSigDB gene sets are curated from 1,329 C2:CP gene sets in MSigDB, by calculating pairwise Jaccard index (Intersection over Union and the Jaccard similarity coefficient), and obtaining pathways with pairwise Jaccard index < = 0.5 (Li et al., 2018).

The sub-network analysis ranks genes and predicts candidates based on InWeb PPI patterns found in known pathways, highlights genes that are more connected to one another than they are to other genes in other modules, and segments them based on their similarity to known pathway gene sets. GeNets also employs a within-degree node-label permutation strategy to build random networks similar to the original network and generate empirical distributions to assess the statistical significance of PPI networks. In addition to InWeb PPI network analysis, GeNets performs gene set enrichment analysis on genes within the network. We performed this function on Molecular Signatures Database (MSigDB) curated Gene Sets (C2, containing pathway databases such KEGG, BioCarta, and Reactome) to test for enrichment of these pathways within the network. The gene set enrichment analysis *p* value is generated based on a hypergeometric test. We used Bonferroni-corrected P < 0.05 to correct for multiple testing.

## Results

### Patients

We identified 274 affected male sib-pairs from multiplex families recruited to Autism Genetic Resource Exchange (AGRE). The mean age difference was 2.5 years between the cohorts of younger and older sibs. General developmental milestones including ages of first word and first walk and cumulative ADI-R scores in the three core domains, e.g., reciprocal social interactions, communication, and repetitive behaviors, were comparable between the cohorts of younger and older sibs (**Supplementary Table 1**).

### Variant Burdens of Sib-Pairs With Severe Versus Mild Deficits in Social Behaviors

Affected sibs (n = 274) were divided into two cohorts with either severe or mild phenotypes defined by total cumulative ADI-R score in three behavioral domains. No significant difference was found for age of diagnosis and general motor development including age of first walk between these two cohorts (**Table 1**). As expected, the total cumulative ADI-R social scores (SOCT_CS) showed a significant difference between these two cohorts (n = 274; SOCT_CS, severe, 24.77 ± 0.27; mild, 17.18 ± 0.43, mean ± SEM; *t*-test; *p* = 1.68E−42) (**Table 1**). A direct comparison of burdens for total cumulative rare (MAF ≤ 0.01), predicted deleterious variants identified no significant difference between these two cohorts (*p* = 0.96).

**Table 1:** Affected Male Sib-Pairs with Severity versus Mild Social Behavioral Deficits.

Affected sibs		Age (Year)	First Walk (Month)	Social behavior (SOCT_CS)
Phenotype	Number	(Mean ± SEM)	(Mean ± SEM)	(Mean ± SEM)
Severe	126	8.53 ± 0.33	12.74 ± 0.21	25.32 ± 0.28
Mild	126	8.15 ± 0.39	12.35 ± 0.15	13.67 ± 0.45
*t*-test		0.67	0.12	2.79E−61

### Variant Burdens of Sib-Pairs With Large Versus Small Differences in Deficits of Social Behaviors

**Figure 1:**
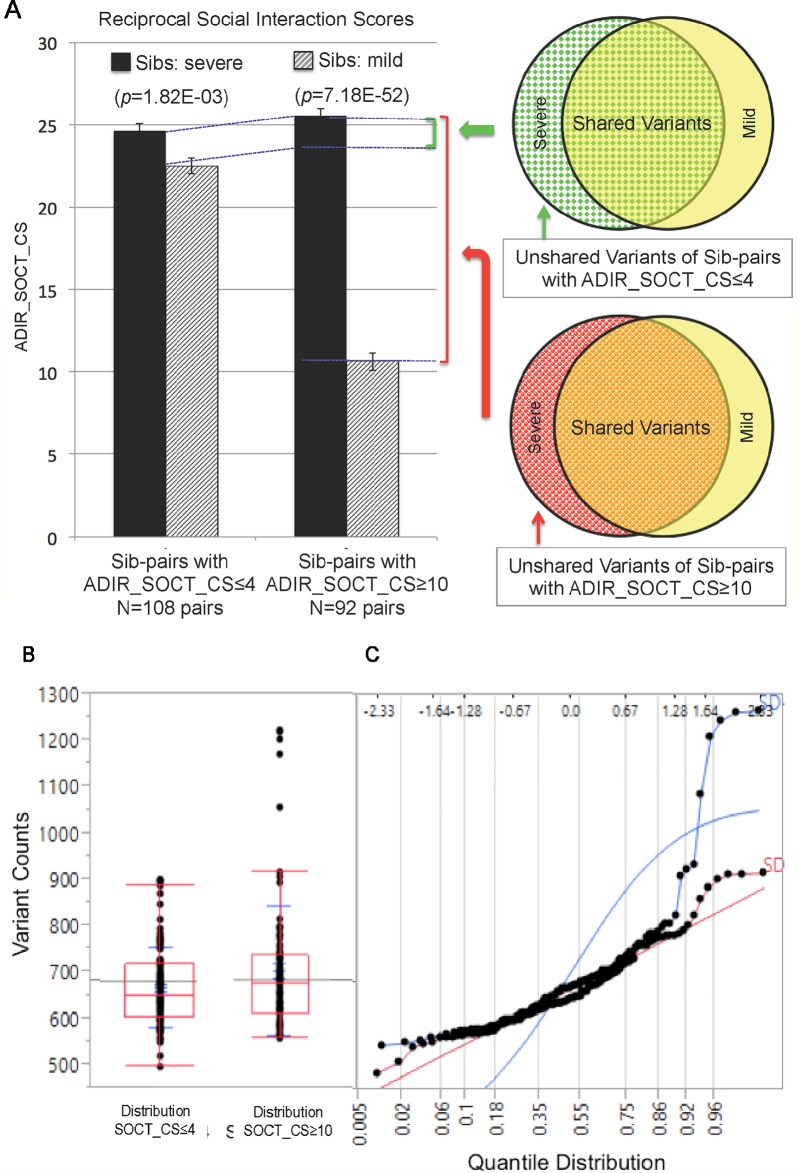
Analysis of Cohorts of Affected Sib-pairs with Large or Small Difference in Severity of Social Behavioral Deficits. **(PanelA)** Comparison of ADIR’s cumulative social behavioral scores (SOCT_CS) between cohorts of affected sib-pairs with either large (SOCT_CS ≥10) or small (SOCT_CS ≤ 4) differences in severity of social behavioral deficits (left); schematic diagram of pools of rare and predicted functional variants for comparison between the affected sibs in these two cohorts (right). **(**Panel **B)** Distribution of rare and predicted functional variants between SOCT_CS≥10 and SOCT_CS ≤ 4 cohorts. **(**Panel **C)** Quantile distribution of rare and predicted functional variants in these two cohorts.

**Figure 2:**
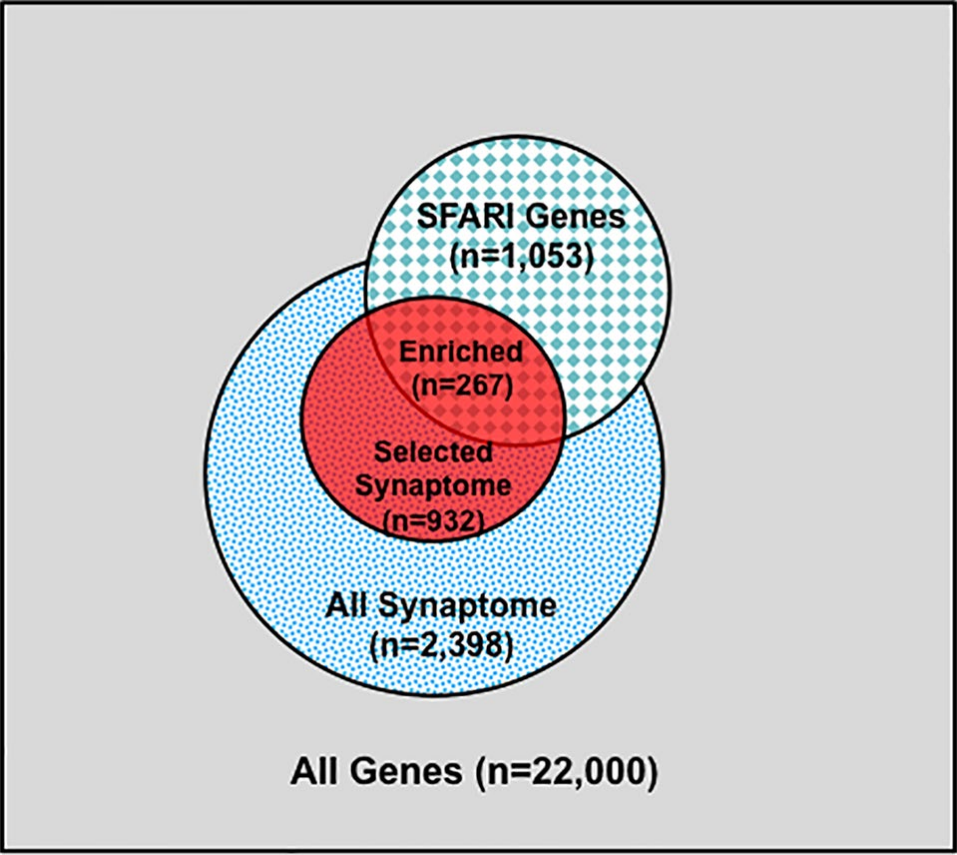
Synaptome Genes with Rare and Predicted Functional Variants are Enriched in SFARI Gene Set. Schematic distribution of total genes (n = 22,000), all synaptome genes (n = 2,398), selected synaptome genes with rare (MAF ≤ 0.01) and predicted functional variants (n = 932), SFARI autism gene set (n = 1,053), and shared genes between the selected synaptome and SFARI gene sets (n = 267).

We next identified subsets of affected sib-pairs with either a large (SOCT_CS≥10, n = 92 pairs; severe, 25.32 ± 0.28; mild, 13.67 ± 0.45; mean ± SEM; *t*-test; *p* = 7.18E−52) or a small difference (SOCT_CS ≤ 4; n = 108 pairs; severe, 24.60 ± 0.44; mild, 22.54 ± 0.47; mean ± SEM; *t*-test; *p* = 1.82E−03) in total cumulative ADI-R social interaction scores between individual sib-pairs (**Table 2 ** and **Supplementary Table 2**). We hypothesized that a larger burden of unshared variants would be seen in the severe only sibs in the SOCT_CS≥10 cohort as compared to that in the SOCT_CS ≤ 4 cohort (**Figure 1A**). We thus extracted variants from each affected sib-pair to identify those unshared variants present only in the sibs with severe social deficits. We compared cumulative allele frequencies and quantile distribution of these sets of unshared variants between the cohorts of SOCT_CS≥10 and SOCT_CS ≤ 4 (**Figures 1B, C**). We observed a higher mutation burden of unshared variants in SOCT_CS≥10 compared to that in SOCT_CS ≤ 4 (SOCT_CS≥10: 705.1 ± 16.2; SOCT_CS ≤ 4, 668.3 ± 9.0; *p* = 0.025), which is consistent with the prior hypothesis. Furthermore, this set of unshared severe only variants in SOCT_CS≥10 are significantly enriched in the SFARI gene set (http://gene.sfari.org) (hypergeometric *p* value: 2.7E−14) (**Figure 2** and **Supplementary Table 3**).

**Table 2:** Affected Sib-Pairs with Large versus Small Difference in Social Behavioral Deficits.

	Large difference (SOCT_CS≥10)		Small difference (SOCT_CS ≤ 4)	
	Age (year)	SOCT_CS*	Age (year)	SOCT_CS*
Sib-pairs (n)	92		108	
Severe	8.8	25.32 ± 0.28	9.29	24.60 ± 0.44
Mild	8.51	13.67 ± 0.45	9.27	22.54 ± 0.47
*t*-test	*p* = 6.17E−1	*p* = 7.18E−52	*p* = 9.71E−1	*p* = 1.82E−03

*ADI-R’s Cumulative Total Social Interaction Score (SOCT_CS).

### Network Analysis to Associate Risk Gene Networks With Social Deficits in Autism

Since these unshared, severe only variants were found by comparing affected sibs in the SOCT_CS≥10 cohort, we speculate that genes harboring these variants are enriched in signaling networks that are crucial to social deficits in autism. To identify signaling genes in networks connected to these variants, we performed a network analyses on the gene-set found in sibs with higher scores in the SOCT_CS≥10 cohort using three platforms (Huguet et al., 2013) InWeb_IM, an integrated human PPI network, (Devlin and Scherer, 2012) MSigDB^(2)^, an annotated gene set for Gene Set Enrichment Analysis (GSEA), and (Gaugler et al., 2014) GeNetMeta, a unified web-based platform for network analyses of genetic data. The InWeb and GeNets algorithm scoring system (Lage et al., 2007) calculates a connectivity *p* value that indicates whether the network was significantly more connected than expected, for a gene set of this size and the global connectivity of its genes, to construct a modular sub-network structure of the underlying genes. The GeNets algorithm builds a general classifier to predict pathway membership from networks in the InWeb PPI network. For any candidate gene, the classifier can assign a probability that it belongs to a pathway as defined by the candidate’s architectural properties in the overall network. This concept will be used to identify functional modules in gene sets and simplify visualizations. A module is a set of genes (called nodes) that are more connected to one another than they are to other groups of genes.

**Figure 3:**
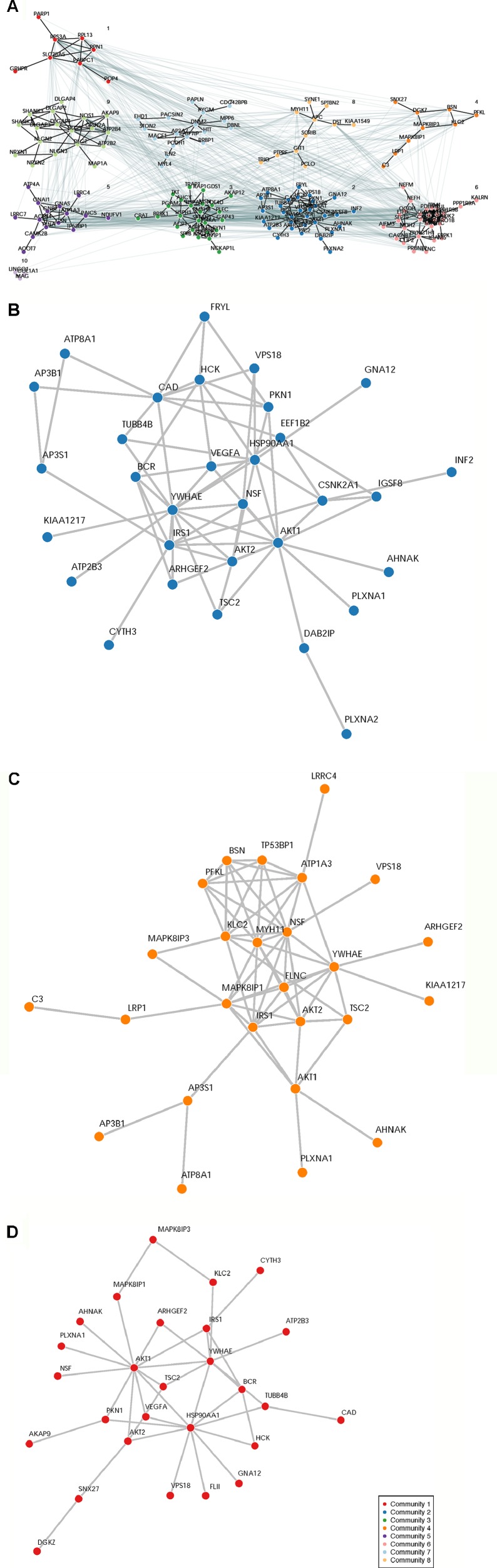
Network Analysis Identified Network Communities Connected to the SOCI_CS≥10 Gene Set. Top 250 synaptome genes that carry rare and predicted functional variants and are shared for ≥3 affected sib-pairs were input separately into the following three analysis platforms to identify connected gene sets and network communities. **(Panel A)** GetNetMeta analysis identified 10 network communities connected to SOCT_CS≥10 geneset. **(Panel B)** SOCT_CS≥10 genes are enriched in community 2 from GeNetsMeta analysis. **(Panel C)** SOCT_CS≥10 genes are enriched in community 4 from InWeb analysis. **(Panel D)** SOCT_CS≥10 genes are enriched in community 1 from MSigDB analysis.

A total of 932 synaptome genes with rare (MAF ≤ 0.01) and predicted functional variants were identified in ≥1 pairs of affected sibs; 276 genes are shared in ≥3 pairs of affected sibs and 32 in ≥10 pairs (**Figure 2** and **Supplementary Table 2**). The top 250 of 276 genes that were shared in ≥3 families were input into these three analysis platforms to identify connected gene sets and communities. Using a connective *p* value of 2.00E−3 as a cutoff, communities enriched for the largest gene sets from each of the three analysis platforms were identified (**Table 3**). GeNetMeta identified 166 connected genes that were classified into 10 communities (**Figure 3A**). Community 2 showed the largest enrichment for network genes (**Figure 3B**). InWeb identified 142 connected genes that were classified into nine network communities (**Supplementary Figure 1**). Community 4 showed the largest enrichment for network genes (**Figure 3C**). MsigDB identified 142 connected genes that were classified into eight network communities (**Supplementary Figure 2**). Community 1 showed the largest enrichment for network genes (**Figure 3D**). A total of 20 seed genes were identified in the communities that showed the largest enrichment from all three platforms (**Table 3**).

**Table 3:** Network Analyses Identify Network Communities Connected to SOCT_CS≥10 Geneset.

Network analysis platform	Input geneset (n)	Connected geneset (n)	Connected network community					Shared Genes (n)
			Total number	Largest geneset	Community size (n)	Enriched genes (n)	Connectivity cutoff (*p*)	
InWeb	250	142	9	4	26	24	2.00E−03	20
MSigDB	250	137	8	1	27	38	2.00E−03	20
GeNetMeta	250	166	10	2	31	25	2.00E−03	20

The sub-network analysis ranks genes and predicts candidates based on InWeb PPI patterns found in known pathways, highlights genes that are more connected to one another than they are to other genes in other modules, and segments them based on their similarity to known pathway gene sets. For significance, the algorithm calculates the density of the network as defined by density = (# of edges/# possible edges) and compute the density for randomly sampled gene sets and its empirical determined *p* value. It computes *p* values for network overall and also by module to test connectivity of these sub-network. Finally, gene set enrichment will be conducted using a Bonferroni-adjusted hypergeometric test on MSigDB canonical pathways. The three analysis platforms identified two top neural signaling networks based on significance, size of connected genes, and their biological relevance (**Table 4**). Eight genes were connected to the phosphoinositide 3-kinase (PI3K)-AKT-tuberous sclerosis complex (TSC)2-mammalian target of rapamycin (mTOR) network with the most significant Enrichment Score (eScore) P value of 3.36E−07. Variants in these eight genes were identified in 68 of 126 patients in the SOCT_CS≥10 cohort. Nine genes were connected to the Nerve growth factor (NGF)-PC12 signaling network with the most significant eScore P value of 8.94E−07. Variants in these nine genes were identified in 50 of 126 patients in the SOCT_CS≥10 cohort. Additional signaling networks of potential importance in autism social behavioral deficits include AVB3-integrin (n = 7 genes), PI3K-ERBB2/4 (n = 4 genes), Sema3A-PKA (n = 3 genes), and Reelin (n = 3 genes) (**Table 5**).

**Table 4:** Two Top Signaling Networks Identified from Gene Communities Connected to SOCT_CS≥10 Geneset.

NEURAL SIGNALING NETWORK	ANALYSIS PLATFORM	eSCORE	OVERLAPING GENES^#^
**PI3K_AKT_MTOR SIGNALING**		
PID_PI3KCI_AKT_PATHWAY	InWeb, MSigDB, GeNetsMeta	2.39E-07	*AKT1, AKT2, HSP90AA1, YWHAE*
ST_PHOSPHOINOSITIDE_3_KINASE_PATHWAY	InWeb, MSigDB, GeNetsMeta	3.01E-07	*AKT1, AKT2, CYTH3, YWHAE*
REACTOME_PI3K_AKT_ACTIVATION	InWeb, MSigDB, GeNetsMeta	3.36E-07	*AKT1, AKT2, IRS1, TSC2*
REACTOME_AKT_PHOSPHORYLATES_TARGETS_CYTOSOL	InWeb, MSigDB, GeNetsMeta	7.35E-07	*AKT1, AKT2, TSC2*
KEGG_MTOR_SIGNALING_PATHWAY	InWeb, MSigDB, GeNetsMeta	1.21E-06	*AKT1, AKT2, TSC2, VEGFA*
PID_MTOR_4PATHWAY	InWeb, MSigDB, GeNetsMeta	3.80E-06	*AKT1, IRS1, TSC2, YWHAE*
REACTOME_PIP3_ACTIVATES_AKT_SIGNALING	InWeb, MSigDB, GeNetsMeta	1.20E-05	*AKT1, AKT2, TSC2*
**NGF-PC12-NEURAL DIFFERENTIATION**			
REACTOME_SIGNALLING_BY_NGF	InWeb, MSigDB, GeNetsMeta	8.94E-07	*AKT1, AKT2, ARHGEF2, IRS1, TSC2, YWHAE*
KEGG_NEUROTROPHIN_SIGNALING_PATHWAY	InWeb, MSigDB	2.02E-05	*AKT1, AKT2, IRS1, YWHAE*
ST_DIFFERENTIATION_PATHWAY_IN_PC12_CELLS	InWeb, MSigDB	2.67E-05	*AKT1, MAPK8IP1, MAPK8IP3*
REACTOME_NGF_SIGNALLING_VIA_TRKA	InWeb, MSigDB	2.80E-05	*AKT1, AKT2, IRS1, TSC2*

^#^AKT1, AKT Serine/Threonine Kinase 1; AKT2, AKT Serine/Threonine Kinase 2; HSP90AA1, Heat Shock Protein 90 Alpha Family Class A Member 1;

YWHAE, Tyrosine 3-Monooxygenase/Tryptophan 5-Monooxygenase Activation Protein Epsilon; CYTH3, Cytohesin 3; IRS1, Insulin Receptor Substrate 1;

TSC2, TSC Complex Subunit 2; ARHGEF2, Rho/Rac Guanine Nucleotide Exchange Factor 2; MAPK8IP1, Mitogen-Activated Protein Kinase 8 Interacting Protein 1;

MAPK8IP3, Mitogen-Activated Protein Kinase 8 Interacting Protein 3.

**Table 5:** Distribution of Shared Genes in Neural Networks from Top Connected Communities.

Gene^#^	Patients No.	PI3A-AKT-mTOR	NGF-PC12	AVB3-INTEGRIN	PI3K-ERBB2/4	SEMA3A-PKA	REELIN
*AKAP9*	3						
*AKT1*	3	+	+	+	+		+
*AKT2*	3	+	+	+	+		
*ARHGEF2*	3		+				+
*BCR*	4						
*CYTH3*	19	+					
*GNA12*	3						
*HSP90AA1*	3	+	+	+		+	
*IRS1*	17	+	+	+	+		
*LRP1*	5						
*MAPK8IP1*	10		+	+			+
*MAPK8IP3*	3		+	+			
*PFKL*	4						
*PKN1*	4						
*PLXNA1*	13					+	
*PLXNA2*	8					+	
*TSC2*	4	+	+		+		
*TUBB4B*	4						
*VEGFA*	9	+		+			
*YWHAE*	4	+	+				
**No. of Genes**		8	9	7	4	3	3
**No. of Patients**		62	50	47	27	24	16
**Total Patients**	126	126	126	126	126	126	126

^#^ AKAP9, A-Kinase Anchoring Protein 9; AKT1, AKT Serine/Threonine Kinase 1; AKT2, AKT Serine/Threonine Kinase 2; ARHGEF2, Rho/Rac Guanine Nucleotide Exchange Factor 2; BCR (Breakpoint Cluster Region); CYTH3, Cytohesin 3; GNA12 (G Protein Subunit Alpha 12); HSP90AA1, Heat Shock Protein 90 Alpha Family Class A Member 1; IRS1 (Insulin Receptor Substrate 1); LRP1 (LDL receptor Related Protein 1): MAPK8IP1, Mitogen-Activated Protein; Kinase 8 Interacting Protein 1; MAPK8IP3, Mitogen-Activated Protein Kinase 8 Interacting Protein 3; PFKL, Phosphofructokinase, Liver Type; PKN1, Protein Kinase N1; PLXNA1, Plexin A1; PLXNA2, Plexin A2; TSC2, TSC Complex Subunit 2; TUBB4B, Tubulin Beta 4B; VEGFA, Vascular Endothelial Growth Factor A; YWHAE, Tyrosine 3-Monooxygenase/Tryptophan 5-Monooxygenase Activation Protein Epsilon. +presence of corresponding genes/proteins in the indicated signaling networks.

## Discussion

We tested a strategy using affected sib-pairs to identify rare genetic variants and signaling networks associated with specific behavioral domains of autism (Hu and Steinberg, 2009; Hu et al., 2011; Sacco et al., 2012; Veatch et al., 2014). This study design explores unshared rather than shared variants and differences in the severity of core behavioral domains between the affected sibs from same-proband families. It is based on the assumptions that: 1) the cumulative rare variants of large effect contribute quantitatively to the observed differences in severity of behavioral domains in autism, and 2) genes carrying these risk variants are clustered in genetic signaling networks associated with deficits in the respective behavioral domains.

Affected sib-pair analysis carries several advantages over traditional designs that study patients from unrelated families. First, affected sibs share a similar environment during prenatal course, infancy, and early childhood. These periods are critical for early brain development and are highly susceptible to the pathogenesis of autism. This approach is expected to minimize environmental influences confounding behavioral phenotypes of autism (Robinson et al., 2014). Genetic factors likely play a bigger role in the observed differences in severity between affected sibs, then between unrelated patients. Second, affected sibs from same families share ≥50% of their genome and presumably, genetic mutations underlying the differences in the severity of specific behavioral domains reside in the unshared portion of genome. Interestingly, a recent WGS study of a cohort of quartet autism families showed that in a large fraction (50–69%) of multiplex ASD families, two affected sibs do not share the same rare penetrant ASD risk alleles (Yuen et al., 2015). Affected sibs with discordant mutations tended to demonstrate more phenotypic variability as compared to those who shared the same risk variants. These results support a strong genetic determinant that is responsible for the difference in severity of the autism phenotype between affected sibs. Third, patients with ASDs manifest a spectrum from mild to severe phenotypes in domains defined by standard behavioral tests. Standard test scores are usually available for patients with a confirmed diagnosis, but not for unaffected relatives. Furthermore, affected sibs enrolled in the AGRE repository were evaluated by the same psychologists using identical sets of behavioral tests, such as the ADI-R, to reduce subjective variations in test scoring. Taken together, our approach effectively enriches genes that regulate autism social behaviors by exploring unshared variants present in severe only sibs of a cohort of affected sib-pairs with large differences in ADI-R social behavioral scores.

It has been suggested that autism-associated mutations cause disturbances in convergent pathways and networks leading to a shared phenotype (Geschwind, 2008). Discovering these common pathways requires a comprehensive, network-based analysis of causal and risk genes (Hormozdiari et al., 2015; Parikshak et al., 2015; Oron and Elliott, 2017). PPI network analysis identifies groups of proteins that physically interact with each other using different databases that curate experimentally validated or predicted PPIs (Parikshak et al., 2015). PPI identifies hubs or highly interconnected proteins, which could be central in the disease-related pathways. The Gene Ontology project provides a unifying description of genes and their biological roles (Ashburner et al., 2000). Using pathway enrichment tools, gene ontology analyses prove to be a highly effective approach to identifying gene networks central to physiological states and disease pathogenesis (Chowdhury and Sarkar, 2015). Network-based analysis has proven to be a powerful approach to identifying risk genes and disease mechanisms in psychiatric disorders with a large genetic contribution.

Two key neural signaling networks, PI3K-AKT-tuberous sclerosis complex (TSC)2-mTOR and NGF-signaling, show significant enrichment for genes harboring unshared variants in SOCT_CS≥10 in connected communities from three analysis platforms in this study. PI3K activates protein kinase B (PKB or AKT), a serine/threonine-specific protein kinase that regulates many aspects of cell physiology including activation of mTOR signaling. In the developing brain, activation of AKT/mTOR signaling is essential for neuronal development, synaptic formation, and plasticity. Increased activity in PI3K-AKT-TSC2-mTOR signaling has been implicated in the syndromic forms of autism including tuberous sclerosis, phosphatase and tensin homolog (PTEN)-related disorders, neurofibromatosis type I, and fragile X syndrome. Inhibition of this increased activity has been shown to improve autism-related symptoms in mouse models of PTEN and TSC1. Using multiple network analysis platforms, our study further implicates a role of this signaling network in the pathogenesis of nonsyndromic autism and particularly in its contribution to social deficits.

Recent studies have implicated NGF signaling in autism core behavioral deficits. Genetic analyses of heritable quantitative traits that correlate with autism identified an association of NGF locus with nonverbal communication in a large cohort of patients (Lu et al., 2013). One study discovered an association of several SNPs in NTRK1 with autism behavioral traits as measured by empathy quotient and autism spectrum quotient (Chakrabarti et al., 2009). Another study on differential alternative splicing in the blood samples from 2- to 4-year-old boys with autism showed a significant difference for several NGF signaling genes including NGF receptor (Stamova et al., 2013). Very interestingly, an animal study on communal nesting (CN), a highly stimulating early social enrichment for rodents, showed that CN results in significant differences in social behaviors later in life and is associated with higher NGF and BDNF levels in the brain of adult mice (Branchi et al., 2006). Additional signaling networks that are also implicated in social behavioral deficits in autism include AVB3-INTEGRIN (Schuch et al., 2014; Dohn et al., 2017; Gabriele et al., 2019), PI3K-ERBB2/4 (Pinto et al., 2010), SEMA3A-PKA, and REELIN signaling (De Rubeis et al., 2014; Lammert et al., 2017; Stessman et al., 2017).

Together, our studies support that multiple signaling networks are involved in the risk and pathogenesis of social behavioral deficits in autism. Analysis of affected sib-pairs shall be a valuable approach to systematically identifying signaling networks crucial to development of the core behaviors in autism spectrum disorders. Comprehensive analyses of genetic variants from whole genome sequencing data, and ADI-R behavioral scores in the three-core domains in an independent cohort of affected sibs with autism, should help to systematically characterize domain-specific and/or overlapping roles for key signaling networks in autism spectrum disorders.

## Data Availability Statement

All datasets for this study are included in the article/the **Supplementary Material**.

## Ethics Statement

The studies involving human participants were reviewed and approved by Johns Hopkins University, IRB-2. Written informed consent to participate in this study was provided by the participants’ legal guardian/next of kin to Autism Genetics Research Exchange (AGRE).

## Author Contributions

MP and TW designed the study. MP, TN, YC-C, IT, and TW performed experiments and/or analyzed data. FG, DA, JP, RH, and PZ contributed reagents and/or data analysis. MP, PZ, and TW wrote the manuscript. All authors read and approved the manuscript.

## Funding

This work was supported in part by research grants from the Simons Foundation Autism Research Initiative (SFARI, #206683], Autism Speaks [#2487], Johns Hopkins Brain Science Institute (BSI), and National Institute of Mental Health, NIH (RO1MH112808).

## Conflict of Interest

The authors declare that the research was conducted in the absence of any commercial or financial relationships that could be construed as a potential conflict of interest.

The handling editor has declared past co-authorship with authors FG, JP, and PZ, as part of the International Consortium on Lithium Genetics (ConLi+Gen) and the Psychiatric Genetics Consortium (PGC).

## Abbreviations

AGRE, autism genetics research exchange; ADI-R, autism diagnostic interview-revised; eScore, enrichment score; SOCT_CS, total cumulative score for social interaction; PPI, protein interaction network; NGF, nerve growth factor; PI3K, phosphoinositide 3-kinase; AKT (PKB), AKT serine/threonine kinase (protein kinase B); TSC, tuberous sclerosis complex; mTOR, mammalian target of rapamycin; PTEN, phosphatase and tensin homolog; CN, communal nesting; MSigDB, molecular signatures database.
