# Supplementary material for: Affected Sib-Pair Analyses Identify Signaling Networks Associated With Social Behavioral Deficits in Autism
Source: Front Genet. 2019 Nov 27;10:1186. doi: 10.3389/fgene.2019.01186 (PMC6892440; doi:10.3389/fgene.2019.01186)
Supplement: Supplementary file 1 [file DataSheet_1.pdf]

Supplemental Table 1. General Development and Autism-related Behaviors of 274 Affected Male Sib-pairs from AGRE

Affected Sibs			General Development		Social behavior	Repetitive Behavior	Communication
Sib Cohort	Number	Age (Year)	First Walk (Mo)	First Word (Mo)	(ADIR_SOCT_CS)	(ADIR_BEHT_CS)	(ADIR_COMVT_CS)
Older	274	9.71 ± 0.27	12.44 ± 0.19	27.15 ± 1.06	21.26 ± 0.43	5.97 ± 0.15	16.62 ± 0.41
Younger	274	7.21 ± 0.25	12.64 ± 0.18	28.30 ± 1.09	20.69 ± 0.34	5.63 ± 0.12	15.92 ± 0.18
t-test		4.28E-11	0.44	0.45	0.34	0.12	0.18

Supplementary Table 2. Phenotypes of Affected Sib-Pairs Cohorts with Large versus Small Differences in Social Behaviors

			Motor and Language Development			Autism Core Phenotype^		
	Sib-Pairs (N)	Age (Year)	First Walk (Month)	First Word (Month)	First Phase (Month)	Social Behavior (SOCT_CS)	Repetitive Behavior (BEHT_CS)	Verbal Communication (COMVT_CS)
SOCT_CS≥10 Cohort								
Severe		8.8±0.36	13.1±0.45	31.2±1.90	50.2±2.25	25.3±0.28	6.7±0.24	18.9±0.41
Mild	92	8.5±0.44	12.4±0.26	21.0±0.26	30.7±1.47	13.7±0.45	4.8±0.31	10.4±0.56
t-test (*p*)		6.23E-01	1.64E-01	4.21E-05	4.71E-08	7.18E-52	1.59E-06	2.47E-17
SOCT_CS≤4 Cohort								
Severe		8.8±0.37	12.4±0.21	30.4±1.96	41.5±2.00	24.60±0.44	6.4±0.22	18.8±0.38
Mild	108	8.2±0.38	12.3±0.25	30.1±1.60	36.7±1.37	22.47±0.47	6.1±0.25	17.6±0.36
t-test (*p*)		2.77E-01	8.45E-01	9.04E-01	1.45E-01	1.06E-03	3.03E-01	1.05E-01

^ SOCT_CS: ADIR total cumulative social behavioral test score; BEHT_CS: ADIR total cumulative repetitive behavioral test score; COMVT_CS: ADIR total cumulative verbal communication test scores

Supplemental Table 3. Synaptome Genes are Enriched in SFARI Autism Gene Set

Affected Sib-Pairs (≥)	Synaptome Gene[#] (No)	SFARI Gene[&] (No)	Odds Ratio	Hypergrometric p-value
1	932	146	2.87	2.70E-14
2	507	94	2.82	2.80E-12
3	276	54	2.61	9.80E-08
4	169	38	2.99	2.90E-07
5	119	29	3.24	1.50E-06
6	86	22	3.39	1.40E-05
7	61	13	2.58	5.00E-03
8	49	13	3.46	5.80E-04
9	42	12	3.83	4.50E-04
10	32	8	3.14	9.90E-03

[#] Number of Synaptome Genes that carry Rare (MAF<1%) and functional variants in affected sib-pairs
[&] Number of SFARI Autism Genes (http://gene.sfari.org) that overlap with above Synamtome genes

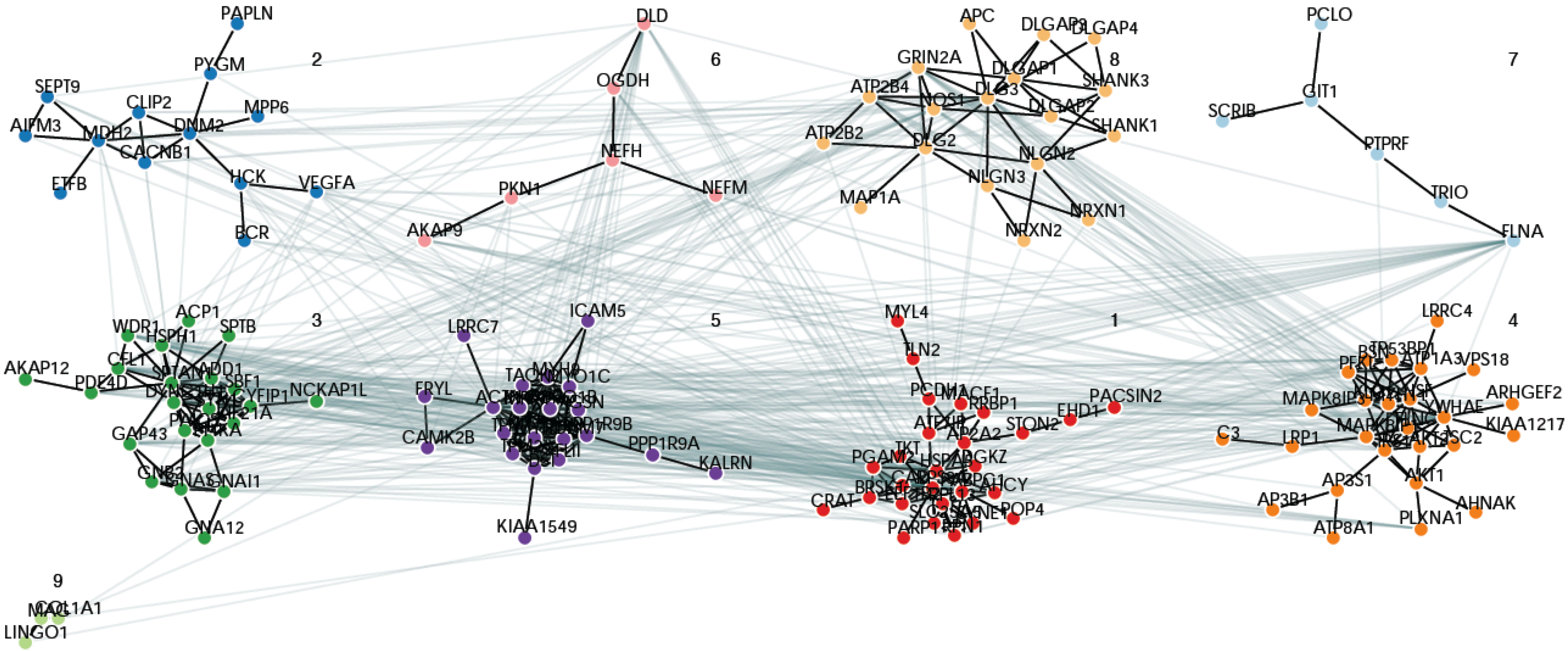

Figure S1. InWeb Analysis Identifies Nine Network Communities Connected to SOCT_CS≥10 Geneset

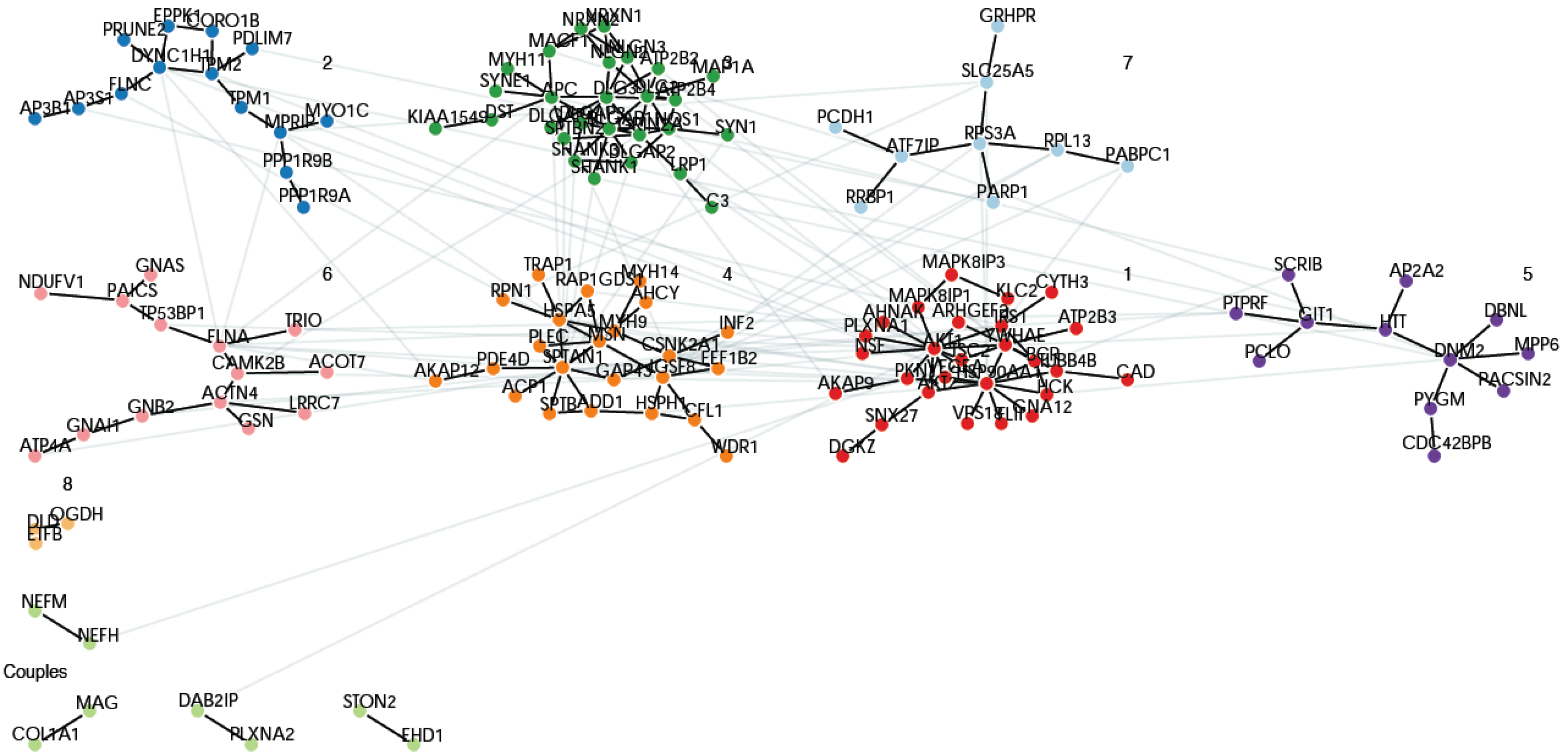

Figure S2. MSigDB Analysis of Identified 8 Network Communities Connected to SOCT_CS≥10 Geneset
